# Isolation and Characterization of Probiotic LAB from Kimchi and Spontaneously Fermented Teff (*Eragrostis tef* (Zucc.) Trotter) Batter: Their Effects on Phenolic Content of Teff during Fermentation

**DOI:** 10.1155/2020/4014969

**Published:** 2020-07-22

**Authors:** Yoseph Asmelash Gebru, Desta Berhe Sbhatu

**Affiliations:** Department of Biological and Chemical Engineering, Mekelle Institute of Technology, Mekelle University, PO Box 1632, Mekelle, Tigrai, Ethiopia

## Abstract

Microbial fermentation is proven to induce molecular transformations and produce bioactive compounds thereby enhancing sensory and nutritional quality of flour-based fermented foods. In this study, lactic acid bacteria (LAB) were isolated from Korean kimchi and Ethiopian fermented teff (*Eragrostis tef* (Zucc.) Trotter) flour batter. Isolates were identified using 16S rRNA gene sequencing and characterized for various probiotic properties. Few trains were selected for further teff flour batter fermentation and evaluating their effects on phenolic contents and compositions. Out of 200 bacterial isolates, 44 of them showed considerable acid and bile tolerance and 22 were tested positive for protease activity. A large number of the isolates showed antimicrobial activities against *Salmonella gallinarium* indicator strains. Majority of these probiotic strains belonged to *Lactobacillus plantarum* and *Lactobacillus brevis* species. All the strains used for fermentation of teff were able to significantly increase total phenolic contents (TPC). An increase in TPC of up to 7-fold was observed in some strains.

## 1. Introduction

Lactic acid bacteria (LAB) are industrially useful, Gram-positive bacteria widely used in food fermentation and probiotic formulations. Probiotics are microorganisms with potential beneficial effects on the health of host organisms [1]. The definition also includes the use of such organisms in the preparations of foods. Interestingly, LABs are the most common types of probiotic microorganisms with a variety of beneficial effects for humans and animals [2]. The most common species are *Lactobacillus acidophilus*, *L. fermentum*, *L*. *casei*, *L*. *brevis*, and *L*. *plantarum.* The potential health benefits are improving immune function, protecting against hostile microorganisms, aiding food digestion and absorption, and producing bioactive compounds that have health benefits. Among the variety of mechanisms by which LAB can confer health promoting effects is the production of bioactive peptides through proteolytic activities in popular probiotic delivery systems such as fermented milk. Bioactive peptides are peptide fragments from food proteins that remain inactive until they are released through hydrolysis to promote a particular physiological effect. For instance, *α*-casein and *β*-casein, the principal milk proteins, can liberate more than 20,000 peptides each on hydrolysis [3]. Similarly, a variety of other microbial enzymes can cleave bonds that hold phenolic compounds and release them to induce their functional properties in food.

LAB fermentation is known to promote hydrolysis of macromolecules and produce bioactive compounds thereby enhancing nutritional quality of flour-based foods such as bread [4]. The flour type can also affect the technological and nutritional qualities of food products and the effectiveness of microbial fermentation [5]. Therefore, it requires selecting suitable starter culture strains for optimizing applications in food preparations. In several previous studies, LAB were mostly isolated from spontaneously fermented common dairy foods [6]. However, it might be more advantageous to isolate LAB strains from a wide range of fermented foods that are claimed to have potential nutritional and functional properties such as Korean kimchi and Ethiopian *injera*.

Teff (*Eragrostis tef* (Zucc.) Trotter) is a small seed (about 0.7% of mass of wheat grain) from Ethiopia mainly used in making pancake-like fermented bread called *injera*. *Injera* is a staple food in Ethiopia and Eritrea and is mainly fermented by LAB [7]. It is also used to prepare several other foods such as cookies and soup. There was no interest in teff in the rest of the world until its nutritional and health benefits were elucidated recently leading to its branding as ‘the new super food'. The increased global demand of teff is the result of its high levels of essential amino acids, gluten-free property, and high mineral contents [8]. The national research council of the USA suggested that teff seeds contain higher amounts of essential amino acids than other cereals and the balance of amino acids is comparable to that of eggs. It is also proven to have a relatively higher phenolic content compared to other common cereals such as wheat. On the other hand, kimchi is a side dish of salted and fermented cabbage with seasoning such as chili powder, spring onion, garlic, and ginger. Several health benefits have been reported for kimchi including improved heart health and a healthy digestive system. The wealth of antioxidants in it exercise healing effects in medical conditions like cancer, diabetes, obesity, atopic dermatitis, and gastric ulcers. This flavonoid and probiotic-rich food delays aging regulates cholesterol levels and boosts the immune system.

Phenolic compounds are present in soluble and bound forms in cereals like wheat and teff. Therefore, the bioaccessibility of significant biochemical transformations occurs to teff bioactive compounds during fermentation resulting in changes of their compositions that, in turn, affect the product property and bioactivity. Changes in teff phenolic profiles during fermentation are important, not only from sensory point of view but also predict potential effects on chronic disease prevention. The aim of this research was to isolate LAB strains with probiotic properties from Korean kimchi and Ethiopian fermented teff batter, identify them to species level using 16s rRNA gene sequencing, screen them for proteolytic activities, and use the selected strains for teff fermentation and evaluate their effects on phenolic contents and compositions.

## 2. Materials and Methods

### 2.1. Chemicals and Reagents

Skim milk, gallic acid, quercetin, and folin-ciocalteu reagent were purchased from Sigma-Aldrich (St. Louis, MO, USA). *Lactobacilli* MRS broth was purchased from Difco Co. (Franklin Lakes, NJ, USA). HPLC grade methanol, acetonitrile, and deionized water were purchased from J.T. Baker Co. (Phillipsburg, NJ, USA). All the other reagents and kits were purchased from different commercial suppliers and were of analytical grade.

### 2.2. Sample Preparation

Five kimchi (cabbage kimchi) samples and 5 fermented teff batter samples were collected from different households in Korea and Ethiopia, respectively. Two teff grain samples (one white and one brown) were collected from local markets in Ethiopia and brought to South Korea with proper packaging. Whole grains were finely ground using a roll mill (single type stainless roller, Shinpoong Eng. Ltd., Gwangju, Gyeonggi, Korea) with 0 mm gap between the rollers and four smashes. Then, the flour was sieved using a 1 mm mesh sieve and stored in the refrigerator at 4°C until use.

### 2.3. Bacterial Isolation, Enumeration and Culture Conditions

Ten (10) grams of kimchi and fermented teff batter samples were prepared separately by mixing with 40 and 60 mL PBS, respectively, and homogenized for 2 min in plastic bags with filter using an automatic bag mixer (BagMixer, InterScience Laboratories, Inc., St. Norn, France). 100 *μ*L from the filtrate compartment of each sample was mixed with 900 *μ*L PBS. Subsequently, appropriate dilutions in PBS were plated on MRS agar. After 18 hrs of incubation at 37 C, microbes were enumerated and predominant LAB colonies were picked. Selection of colonies was based on diversification of colony morphology so as to include all different colony types as much as possible. Then, each strain was purified through successive streaking on fresh MRS agar before culturing in MRS broth for further experiments. Finally, isolated strains were stored at –80 C with 30% glycerol at a concentration of above 10^6^ cfu/mL.

### 2.4. Identification of Strains through 16S rRNA Gene Sequencing

Bacterial genome was extracted from 1 mL 18 hrs old culture using GeneAll Exgene Cell SV kit (GeneAll Biotechnology Co. Ltd., Seoul, Korea), and the 16S rRNA gene was amplified by PCR. The universal primers 27F (5′-AGAGTTTGATCMTGGCTCAG-3′) and 1492R (5′-TACGGYTACCTTGTTACGACTT-3′) were used for the PCR amplification of the 16S rRNA gene. Then, PCR products were purified using HiYield Gel/PCR DNA Mini kit (Real Biotech Corporation, Minsheng Rd, Banqiao City, Taiwan) after separating them electrophoreticaly at 50 V on 0.8% (w/v) agarose gel. Molecular sizes of the PCR products were estimated by comparison with 1 kb DNA marker (BioNeer, Munpyeongseo Rd., Daejeon, Korea). Nucleotide sequences were determined at Macrogene Korea (Beotkkot Rd, Seoul, Korea) through cycle extension in an ABI 373 DNA sequencer (Applied Biosystems, Foster City, CA, USA). The sequencing results of each primer were imported to the BioEdit software program for assembly and alignment to generate reliable contigs after editing and removing ambiguous sequences. Finally, sequence similarities of each contig were examined by searching their homologies in the GeneBank database using BLAST. The hits after analyses were used to identify each isolate at a species level.

### 2.5. Determination of Proteolysis Activity of Isolates

Candidate strains were screened for proteolytic activity using casein as a substrate. Overnight cultures (18 hrs old) of strains were centrifuged at 13,000 rcf for 10 min at 4°C. Then, the supernatant was used for the enzymatic assay described as follows. A 20 *μ*L of culture supernatant was dotted on a 6 mm nitrocellulose disc previously placed on skimmed milk agar (final composition of 1% skim milk, 1.5% agar) and air dried for 30 min. Finally, plates were inverted and incubated at 37°C overnight (18 hrs). The diameters of clear zones around the discs were measured to estimate proteolytic activity.

### 2.6. Screening for Survival under Gastrointestinal Tract (GIT) Conditions

Each isolated strain was tested for acid and bile salt tolerance as follows. For acid tolerance assay, purified bacterial colonies were inoculated in MRS broth (pH 6.5) and incubated at 37°C for 18 hrs. Then, a fresh MRS broth was inoculated with 1% of the overnight culture and reincubated overnight (18 hrs) to balance growth phase (late exponential). To measure their survival in acidity, 1 mL of overnight culture was transferred to 9 mL PBS (pH adjusted to 2.5 with 2 N HCl) and incubated at 37°C shaking incubator. The number of viable bacteria was counted at 0 and 3 hrs of incubation by plating on MRS agar. Experiments were done in triplicate and the mean survival rates were calculated.

For the bile salt tolerance assay, purified colonies were inoculated into MRS broth and incubated at 37°C for 18 hrs. Then, a fresh MRS broth was inoculated with 1% overnight culture and reincubated overnight (18 hrs) to balance growth phase (late exponential). To measure their survival in bile salts, 1 mL of overnight culture was transferred to 9 mL MRS broth containing 0.3% (*w*/*v*) bile salts (cholic acid sodium salt 50%, deoxycholic acid sodium salt 50%), Sigma) and incubated at 37°C shaking incubator. Finally, viable bacteria were enumerated at 0 and 24 hrs of incubation time by plating on MRS agar. Experiments were done in triplicate and the mean survival rates were calculated.

### 2.7. Antimicrobial Activity Assay

Antimicrobial activity was examined by well diffusion assay (WDA) against eight food born pathogenic indicator strains—four *Salmonella enteritidis* (HJL344, HJL349, HJL377, and HJL385) and four *Salmonella gallinarium* (HJL467, HJL482, HJL510, HJL517—obtained from the Department of Veterinary Science, Chonbuk National University, Iksan campus. Determination of antimicrobial activity was conducted as follows. MRS broth was inoculated with LAB strains and incubated for 24 hrs at 37°C. Extracellular cell free supernatants (ECFS) from these cultures were collected by centrifugation (8,500 rcf for 10 min). The pH of the ECFS were measured and divided in to two equal parts. The first part was left acidic and the second was neutralized with 6 N NaOH to pH 6.0. All the supernatants were filter sterilized. A 100 *μ*L overnight culture of indicator strains was spread on TSB agar and an 8 mm by 5 mm well was made on the agar using cork borer. Then, 10–20 *μ*L supernatants were added to the wells and incubated for 10 hrs. Finally, the diameters of inhibition zones around the wells were measured.

### 2.8. Fermentation of Teff Batter

Teff fermentation was conducted as described by Fischer et al. [7] with some modifications. For the lab-scale fermentation of teff flour, each LAB strain was grown overnight in MRS broth. Each cell culture was plated to measure and calculate cfu/mL based on a previous standard curve prepared for each isolate. Teff flour was autoclaved by spreading it on aluminum foil. A 9.6 g sterile tap water and 0.4 mL peptone solution containing the respective starter cultures at 10^7^–10^8^ cfu/g and 6 g flour were mixed in 100 mL bottles. Then, samples were incubated at 37°C. As control, teff flour suspension was held under the same conditions without starter cultures. Three types of samples (0, 24, and 48 hrs of fermentations) were used for analysis. At the end of the fermentation, batter samples were freeze dried and stored at 4°C until use.

### 2.9. Extraction of Soluble Phenolic Compounds

Extraction of soluble phenolic compounds was performed as per the method described by Shumoy and Raes [9] with some modifications. One (1) g teff flour was extracted three times with 80% aqueous methanol at 1 : 5 flour/solvent ratio by shaking at 200 rpm for 1 hr followed by 30 min of sonication (Ultrasonicator, Hwa Shin Instrument Co., Seoul, Korea) at room temperature (RT). Then, the extraction mixture was centrifuged at 4,500 cfr (VS-550, Vision Scientific, Daejon, South Korea) for 15 min and all supernatants from the three extractions were combined and transferred to new tubes. Finally, the extracts were filtered through 0.2 *μ*m membrane filters (Roshi Kaisha, Tokyo, Japan) and stored in refrigerator at –20°C until use.

### 2.10. Determination of Total Phenol

Total phenolic contents (TPC) of samples were determined according to the method described by Chandra et al. [10] with some modifications. A 200 *μ*L methanol extract was mixed with 1 mL of 1 N sodium carbonate, vortexed briefly, and incubated for 2 min at RT. Then, 800 *μ*L of folin–ciocalteu phenol reagent was added, and the mixture was vortexed for 10 sec. After incubation for 30 min at RT in dark condition, absorbance was read at 720 nm using UV-visible spectrophotometer (Biochrom–Libra S22, Cambridge, UK) against a blank (80% methanol). The TPC was calculated based on a calibration curve of gallic acid. Results were expressed as mg of gallic acid equivalent per 100 g of grain flour (mg GAE/100 g dw).

### 2.11. Determination of Total Flavonoid

Total flavonoid contents (TFC) were determined as per the method described by Zhishen et al. [11] with slight modifications. A 75 *μ*L of 5% sodium nitrite solution was added into 250 *μ*L MeOH extract of each sample and vortexed briefly. After 5 min of incubation at RT, 10% aluminum chloride was added and the mixture was vortexed. Then, the reaction mixture was incubated for 6 min at RT and 500 *μ*L of 1 N NaOH was added. Finally, the total volume was adjusted to 1 mL with distilled water and absorbance was measured at 510 nm against a blank (80% methanol). The TFC was calculated using a calibration curve of quercetin and results were expressed as mg quercetin equivalent (QE) per 100 g of flour sample (mg QE/100 g dw).

### 2.12. Statistical Analyses

All experiments were performed in triplicate and results were expressed as mean with standard deviation (mean ± SD). Statistical analyses were conducted with GraphPad Prism 5 (GraphPad Software, San Diego, CA, USA) for Windows and a one-way analysis of variance (ANOVA). Duncan's multiple range tests were carried out to test any significant difference in the phenolic contents before and after fermentations. Values with *p* ≤ 0.05, fixed *a priori*, were considered significantly different.

## 3. Results and Discussions

About 200 LAB were isolated from five kimchi and four fermented teff batter samples to use them for fermentation of teff flour, thereby exploring the phenolic changes they bring about in fermented teff batter. Then, 167 isolates that showed good growth on MRS agar were screened for primary probiotic properties, mainly acid and bile tolerance. Of these, 44 isolates were able to show considerable acid and bile tolerance. The tolerant isolates were used to investigate their proteolytic and antimicrobial activities. Twenty-two (22) isolates were tested positive for protease activity out of which seven were selected for fermentation of teff and evaluations of phenolics contents after fermentation. Results of the studies are presented below.

### 3.1. Bacterial Growth and Enumeration

The microbial count after plating on MRS agar (pH 6.5) for each isolate from the kimchi and teff batter samples ranged from 10^5^ to 10^9^ cfu/g with an average of about 10^6^ cfu/g. A total of 200 strains were isolated from 11 samples (from six kimchi, one fermented food factory wastewater, and four fermented teff batter samples). All the kimchi and wastewater isolates showed good growth in MRS broth under aerobic incubation whereas all samples isolated from the teff flour batter could only grow well under microaerobic condition of tightly capped falcon tubes [12].

### 3.2. Survival of Isolates under GIT Conditions

Of the 200 LAB isolates, the 44 isolates that showed considerable acid and bile salt tolerance were selected for further tests. The survival rates during acid and bile tolerance screening were calculated as follows (where stress represents MRS media with bile salts or pH 2.5):

(1) $$\begin{array}{c} \text{Survival Rate }\left(\%\right)=\log{\text{CFU}}_{\text{With Stress}}\times 100/\log{\text{CFU}}_{\text{Without Stress}}. \end{array}$$

It can be seen from the results that most of the selected isolates showed significant acid and bile tolerance (Table 1). About 0.3% bile salts concentration has been considered to be the critical concentration used in the selection of resistant strains for probiotic preparations [13]. It is also well established that the pH of the gastric environment is known to be 2.5 [14]. Previous studies reported similar levels of acid and bile salt tolerance of LAB isolates such as *Lactobacillus plantarum* strains [15].

### 3.3. Identification of Selected Strains through 16S rRNA Gene Sequencing

All the isolates that showed either acid or bile tolerance were screened for proteolytic activity. Twenty-two (22) of them were positive for protease activity. Then, the 22 isolates were identified at species level using 16S rRNA gene sequencing. Ten (10) isolates were identified as *Lactobacillus brevis*, nine (9) isolates were identified as *Lactobacillus plantarum*, and two (2) were identified as *Bacillus amyloliquefaciens* strains (Table 2). Additionally, one (1) isolate was identified as *Bacillus velezensis* strain.

### 3.4. Proteolytic Activity of Isolates

The proteolytic activities of the identified strains were determined by skim milk agar assay and were expressed as diameter of halos (clear zones) formed after the degradation of casein by extracellular proteases secreted from or by the isolates. Among the 22 isolated strains tested for proteolytic activity, K14 (Lactobacillus plantarum) and B31 (Bacillus amyloliquefaciens) produced clear zones with bigger diameters (Table 3). Extracellular proteases secreted from LAB hydrolyze the peptide bonds in the casein protein in skim milk and therefore break it down either into smaller peptides or amino acid constituents [16]. Microbial proteases play vital roles in several industrial applications such as leather processing, meat processing and cheese making. In this study, the isolates that showed relatively higher proteolytic activities were used for the fermentation of teff batter in an attempt to enhance its functional properties, particularly phenolic content.

### 3.5. Antimicrobial Activity of Isolates

The most important probiotic property of LAB is their ability to inhibit the growth of pathogenic bacteria. The pathogenic bacteria used as indicator strains in this study were four *Salmonella enteritidis* strains (HJL344, HJL349, HJL377, and HJL385) and four *Salmonella gallinarium* strains (HJL467, HJL482, HJL510, and HJL517). The results showed that most of the isolates have antimicrobial activities against the *S*. *gallinarium* indicator strains. The *S*. *enteritidis* seemed to show some tolerance (Table 4). The inhibitory properties of LAB isolates could be due to the H_2_O_2_, organic acids, and specific bacteriocins they produce [17, 18].

### 3.6. Changes in Phenolic Contents of Teff during Fermentation with LAB

**Figure 1:**
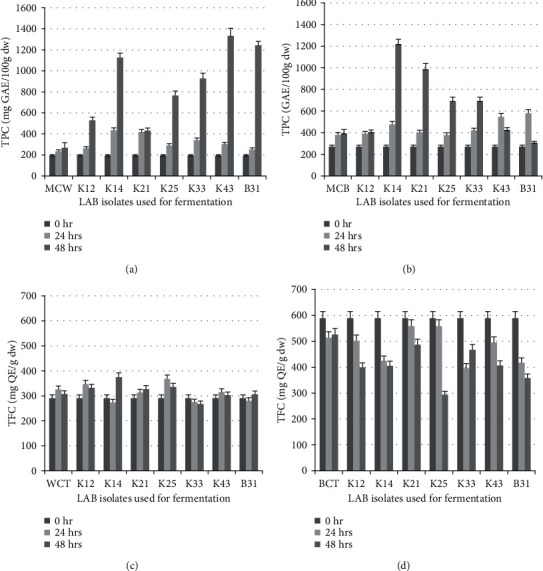
Changes in phenolic and flavonoid contents of teff during fermentation with lactic acid bacteria. (a) TPC in white teff; (b) TPC in brown teff; (c) TFC in white teff; (d) TFC in brown teff. MCW: matched control white teff; MCB: matched contol brown teff; WCT: white teff contol; BCT: brown teff control.

Out of the 22 isolates that tested positive for protease activity, seven (7) of them were selected to be used as starter cultures for white and brown teff fermentations. The effect of fermentation on phenolic content of teff batter was monitored at 24 and 48 hrs fermentation times. The results of changes in TPC and TFC after fermentation with each strain are presented in Figure 1 (refer also to Table 5).

All isolates were able to increase TPC in both grain types (Figures 1(a) and 1(b)). However, the effects of the fermentation on phenolic contents showed noticeable differences between the white and brown teff samples as well as due to fermentation time. Generally, increasing the fermentation time from 24 to 48 hrs significantly increased the phenolic content. Exceptions were isolates of K43 and B31 that gave higher TPC in the brown teff samples in 24 hrs fermentation. These result could have been caused by the growth patterns of the isolates or the stability of their proteases after secretion into the media. This can be confirmed by testing at different time intervals but we cannot conduct that experiment at this time. On the other hand, K21 (in white teff samples) and K12 isolates (in brown teff samples) did not show significant difference due to fermentation time. The highest (7-fold) increase in TPC of white teff was observed in K43 isolate in 48 hrs fermentation (1,331.40 ± 56.32 mg GAE/100 g) followed by B31 (6.5-fold increase), K14 (6-fold increase), K33 (5-fold increase), and K25 (about 4-fold increase) compared with the nonfermented sample. In the case of brown teff, 48 hrs fermentation with isolate K14 gave the highest increase (4.5-fold) in TPC followed by isolate K21 and isolates K25 and K33 that yielded 3.6-fold and 2.5-fold increase in TPC, respectively.

Increments in TPC contents were reported for some cereals such as rye, barley, and oat after fermentation with LAB [19, 20]. However, there is no previous empirical data on the effects of LAB fermentation on teff phenolics. LAB contain various sets of enzymes that can transform phenolic compounds. Extracellular enzymes of LAB are known to hydrolyze complex polyphenols and their glycosylated compounds into free forms of phenolic acids during sourdough fermentations [21, 22]. More than 20-fold increase in phenolic contents were recorded in fermentation of barely and oat with probiotic LAB such *L*. *casei*, *L*. *brevis*, and *L*. *plantarum* strains. In a study that examined the effects of fermentation on red sorghum dough reported an increase in phenolic contents through release of bound phenolic compounds, partial hydrolysis of glycerol esters of phenolic compounds, and partial conversion of flavonoid hexosides into the corresponding flavonoids [23]. The difference in the patterns of phenolic changes between white and brown teff may be accounted to the genetic variations between the two grain types. There are different sets of indigenous enzymes that may contribute to hydrolysis of polyphenols in the two grain types. Moreover, molecules that may induce or suppress LAB enzymes may also contributed to the different patterns in phenolic changes.

Changes in TFC also showed marked differences between grain types. Only few isolates showed slight increases of TFC in the white teff while all the brown samples showed sever reductions after fermentation with every isolate (Figures 1(c) and 1(d)). The most significant reduction in TFC was observed in isolate K25 where it decreased the TFC by almost half after 48 hrs of fermentation compared with the control (Figure 1(d)). This indicates that as the fermentation progressed, hydrolysis of the flavonoid compounds by enzymes released by the starter culture increased. Flavonoid compounds were reported to be converted to smaller molecules such as phenolic acids and other metabolites by bacterial enzymes [24]. The difference between the white and brown teff samples is obvious considering their genetic variations and the higher initial flavonoid content in the brown one. Flavonoids are major group of dietary phenolic compounds [25]. Some studies consider hydrolysis of flavonoids by probiotic bacteria as a good outcome. Prior metabolism of flavonoids is very useful as they are poorly absorbed in the gut. Gut microbiota are known to metabolize flavonoids so that their metabolites reach circulation system and exert their anti-inflammatory effects [26].

## 4. Concluding Remarks

This study isolated LAB from Korean kimchi and spontaneously fermented Ethiopian teff flour batter and screened them for probiotic characteristics. A major fraction of the isolates showed considerable acid and bile tolerance. Many isolates from the acid and bile tolerant groups also showed significant antimicrobial activities, especially against the *S. enteritidis* indicator strains. Isolates with good protease activities were selected for teff fermentation to examine how fermentation affects phenolic contents. Fermentation using the isolates caused significant increase in TPC in teff. But the reverse was true for TFC where fermentation with majority of the isolates lowered the TFC content. In conclusion, fermentation of teff flour batter with a suitable starter LAB can enhance its functional property by increasing the contents of phenolic acids and enhancing the bioavailability of flavonoids.

## Figures and Tables

**Table 1:** Acid and bile salt tolerance of LAB isolated from kimchi and fermented teff batter.

Isolates	Mean (SD) survival rates (%)	
	pH 2.5; 3 hrs	0.3% bile salts; 24 hrs
K11	12.92 ± 1.21	74.10 ± 6.93
K12	17.75 ± 4.66	72.90 ± 6.82
K13	19.01 ± 3.78	69.76 ± 6.52
K14	66.73 ± 6.24	39.09 ± 3.66
K15	57.19 ± 5.35	73.96 ± 6.92
K16	16.01 ± 1.50	86.00 ± 8.04
K17	53.48 ± 5.00	81.37 ± 7.61
K18	19.20 ± 1.80	27.59 ± 2.58
K21	53.59 ± 5.01	70.99 ± 6.64
K22	37.41 ± 3.50	66.48 ± 6.22
K25	32.67 ± 3.06	69.49 ± 6.50
K31	78.21 ± 7.31	73.44 ± 6.87
K33	77.85 ± 7.28	47.12 ± 4.41
K34	65.31 ± 6.11	70.60 ± 6.60
K35	47.04 ± 4.40	77.58 ± 7.26
K36	65.82 ± 6.16	70.64 ± 6.61
K37	56.19 ± 5.25	73.38 ± 7.86
K42	34.00 ± 3.18	71.86 ± 6.72
K43	58.13 ± 5.44	54.32 ± 5.08
K44	68.03 ± 6.36	72.49 ± 6.78
K45	43.76 ± 4.09	71.51 ± 6.69
K47	43.33 ± 4.05	59.89 ± 5.60
K48	47.39 ± 9.43	56.71 ± 5.30
K49	49.25 ± 4.61	71.94 ± 6.73
S16	68.02 ± 6.36	72.65 ± 12.79
B11	76.59 ± 7.16	90.98 ± 14.75
B12	80.67 ± 11.54	92.30 ± 6.42
B13	75.22 ± 7.03	88.65 ± 5.77
B14	79.58 ± 8.44	90.17 ± 11.79
B15	81.58 ± 7.63	92.77 ± 5.97
B16	69.62 ± 6.51	92.10 ± 9.14
B17	67.83 ± 6.34	90.42 ± 8.74
B18	76.34 ± 7.14	89.51 ± 10.06
B19	72.95 ± 6.82	93.22 ± 6.45
B110	75.14 ± 7.03	89.94 ± 6.35
B21	82.52 ± 9.72	87.32 ± 8.77
B22	76.41 ± 10.15	87.72 ± 6.56
B23	83.16 ± 7.78	93.15 ± 6.00
B24	74.26 ± 6.94	89.76 ± 11.46
B26	73.98 ± 6.92	94.03 ± 8.71
B27	83.78 ± 7.84	88.16 ± 11.03
B28	75.38 ± 7.05	89.21 ± 8.40
B29	81.42 ± 7.61	87.22 ± 9.77
B210	75.99 ± 8.65	88.95 ± 4.17

Isolate labels starting with ‘K' belongs to those isolates obtained from kimchi. Isolate label starting with ‘S' belongs to those isolates obtained from fermented food factory wastewater. Isolate labels staring with ‘B' belongs to those isolates obtained from fermented teff batter.

**Table 2:** Identities of LAB isolates as determined by 16s rRNA gene sequencing.

Isolates	Sources	Identification (species)
K11	Kimchi	*Lactobacillus plantarum*
K14	Kimchi	*Lactobacillus plantarum*
K12	Kimchi	*Lactobacillus brevis*
K13	Kimchi	*Lactobacillus brevis*
K15	Kimchi	*Lactobacillus brevis*
K17	Kimchi	*Lactobacillus plantarum*
K18	Kimchi	*Lactobacillus plantarum*
K21	Kimchi	*Lactobacillus brevis*
K22	Kimchi	*Lactobacillus brevis*
K25	Kimchi	*Lactobacillus plantarum*
K31	Kimchi	*Lactobacillus plantarum*
K33	Kimchi	*Lactobacillus plantarum*
K34	Kimchi	*Lactobacillus brevis*
K35	Kimchi	*Lactobacillus brevis*
K36	Kimchi	*Lactobacillus brevis*
K37	Kimchi	*Lactobacillus plantarum*
K42	Kimchi	*Lactobacillus brevis*
K43	Kimchi	*Lactobacillus brevis*
K45	Kimchi	*Lactobacillus plantarum*
B16	Teff batter	*Bacillus velezensis*
B31	Teff batter	*Bacillus amyloliquefaciens*
B19	Teff batter	*Bacillus amyloliquefaciens*

Isolate labels starting with ‘K' belongs to those isolates obtained from Kimchi. Isolate labels staring with ‘B' belongs to those isolates obtained from fermented teff batter.

**Table 3:** Proteolytic activity of LAB isolates.

Isolates	Diameter of halo (mm)
K11	16.33 ± 1.53
K14	18.13 ± 1.73
K12	17.67 ± 1.15
K13	13.67 ± 1.06
K15	14.00 ± 2.06
K17	14.84 ± 2.13
K18	15.36 ± 2.08
K21	16.73 ± 1.53
K22	15.41 ± 2.65
K25	17.00 ± 2.07
K31	16.00 ± 0.58
K33	16.50 ± 2.31
K34	14.33 ± 0.58
K35	14.67 ± 1.00
K36	14.00 ± 2.08
K37	16.67 ± 2.65
K42	16.00 ± 1.00
K43	17.30 ± 2.08
K45	16.33 ± 0.67
B16	15.02 ± 1.43
B31	18.50 ± 1.53
B19	13.42 ± 1.15

Values are mean ± SD; *n* = 3. Isolate labels starting with ‘K' belongs to those isolates obtained from Kimchi. Isolate labels staring with ‘B' belongs to those isolates obtained from fermented teff batter.

**Table 4:** Antimicrobial activity of CFS of LAB isolates against Salmonella sp. indicator strains.

Isolates	Diameter of inhibition Ione (mm) of Indicator strains							
	HJL344	HJL349	HJL377	HJL385	HJL467	HJL482	HJL510	HJL517
K11	+	++	—	—	++	—	++	—
K14	++	+++	—	—	+	—	—	—
K12	—	+++	—	—	—	—	—	—
K13	+	+++	—	—	—	—	—	—
K15	++	+	—	—	—	—	—	—
K17	++	+++	—	—	—	—	—	—
K18	++	+++	—	—	+	—	—	—
K21	++	+++	—	—	—	—	—	—
K22	+++	+++	—	—	—	—	—	—
K25	+++	+++	—	—	—	—	—	—
K31	+++	+++	—	—	—	—	—	—
K33	+	+++	+++	+++	+++	—	+++	+
K34	+	+++	+++	+++	+++	—	+++	+
K35	++	+++	+++	+++	+++	—	+++	+
K36	++	+++	++	+++	+++	—	+++	+
K37	++	+++	+++	+++	+++	—	+++	+
K42	—	—	+++	+++	++	—	—	—
K43	—	—	+++	+++	—	—	+	—
K45	—	+++	+++	+++	—	—	+	—
B16	—	+++	+++	+++	—	—	—	—
B31	—	—	—	—	—	—	—	—
B19	—	—	—	—	—	—	—	—

Symbols for diameter of zone inhibition. +++: >20 mm. ++: 19 to 15 mm. +: 14 to 10 mm.

**Table:** (a) Total phenolic contents

Teff variety	LAB isolate	Mean (SD) TPC (mg GAE/100 g dw) of fermented teff batter		
		0 hr	24 hrs	48 hrs
White	MCW	191.94 (10.75)^a^	235.20 (13.17)^a^	268.14 (48.62)^a^
	K12	191.94 (10.75)^a^	264.18 (14.79)^ab^	529.62 (29.66)^c^
	K14	191.94 (10.75)^a^	434.28 (24.32)^e^	1,126.02 (43.06)^f^
	K21	191.94 (10.75)^a^	418.32 (23.43)^e^	431.76 (24.18)^b^
	K25	191.94 (10.75)^a^	291.06 (16.30)^bc^	765.66 (42.88)^d^
	K33	191.94 (10.75)^a^	341.46 (19.12)^d^	926.10 (51.86)^e^
	K43	191.94 (10.75)^a^	302.82 (16.96)^c^	1,331.40 (74.56)^h^
	B31	191.94 (10.75)^a^	253.26 (14.18)^a^	1,241.52 (39.53)^g^
Brown	MCB	270.06 (15.12)^a^	380.52 (21.31)^a^	389.98 (39.84)^b^
	K12	270.06 (15.12)^a^	388.92 (21.78)^a^	401.94 (22.51)^b^
	K14	270.06 (15.12)^a^	476.70 (26.7)^b^	1,217.16 (48.16)^e^
	K21	270.06 (15.12)^a^	401.10 (22.47)^a^	984.06 (55.11)^d^
	K25	270.06 (15.12)^a^	377.58 (21.14)^a^	689.22 (38.60)^c^
	K33	270.06 (15.12)^a^	419.16 (23.47)^a^	690.06 (38.64)^c^
	K43	270.06 (15.12)^a^	547.26 (30.65)^c^	422.94 (23.68)^b^
	B31	270.06 (15.12)^a^	580.44 (32.50)^c^	303.66 (17.00)^a^

Means in the same column with different letters are statistically significantly different at *p* ≤ 0.05.

**Table:** (b) Total flavonoid contents

Teff variety	LAB isolate	Mean (SD) TFC (mg GAE/100 g dw) of fermented teff batter		
		0 hr	24 hrs	48 hrs
White	MCW	291.26 (12.82)^a^	325.04 (14.30)^bc^	306.78 (13.50)^bc^
	K12	291.26 (12.82)^a^	346.96 (15.27)^cd^	331.43 (14.58)^cd^
	K14	291.26 (12.82)^a^	273.91 (12.05)^a^	375.26 (16.51)^e^
	K21	291.26 (12.82)^a^	312.26 (13.74)^b^	326.87 (14.38)^bcd^
	K25	291.26 (12.82)^a^	367.04 (16.15)^d^	335.09 (14.74)^d^
	K33	291.26 (12.82)^a^	274.83 (12.09)^a^	267.52 (11.77)^a^
	K43	291.26 (12.82)^a^	314.09 (13.82)^b^	302.22 (13.30)^b^
	B31	291.26 (12.82)^a^	280.30 (12.33)^a^	305.87 (13.46)^bc^
Brown	MCB	588.91 (25.91)^a^	514.04 (22.62)^b^	525.91 (23.14)^e^
	K12	588.91 (25.91)^a^	502.17 (22.10)^b^	399.00 (17.56)^c^
	K14	588.91 (25.91)^a^	424.57 (18.68)^a^	405.39 (17.84)^c^
	K21	588.91 (25.91)^a^	558.78 (24.59)^c^	486.65 (21.41)^d^
	K25	588.91 (25.91)^a^	558.78 (24.59)^c^	294.00 (12.94)^a^
	K33	588.91 (25.91)^a^	396.26 (17.44)^a^	466.57 (20.53)^d^
	K43	588.91 (25.91)^a^	494.87 (21.77)^b^	406.30 (17.88)^c^
	B31	588.91 (25.91)^a^	417.26 (18.36)^a^	357.91 (15.75)^b^

Means in the same column with different letters are statistically significantly different at *p* ≤ 0.05.

## Data Availability

The data used to support this study can be obtained from the first author upon request.
